# The Impact of Location on De Novo Spondylodiscitis: Regions Matter but Are Secondary to Comorbidities

**DOI:** 10.3390/jcm14103303

**Published:** 2025-05-09

**Authors:** Julius Gerstmeyer, Anna Gorbacheva, Clifford Pierre, Mark Kraemer, Colin Gold, Cameron Hogsett, Nick Minissale, Periklis Godolias, Tobias L. Schulte, Thomas A. Schildhauer, Amir Abdul-Jabbar, Rod J. Oskouian, Jens R. Chapman

**Affiliations:** 1 Swedish Neuroscience Institute, Swedish Medical Center, Seattle, WA 98122, USA; 2 Department of Orthopedics and Trauma Surgery, St. Josef Hospital Bochum, Gudrunstraße 56, 44791 Bochum, Germany; 3 Department of General and Trauma Surgery, BG University Hospital Bergmannsheil, Ruhr University Bochum, Bürkle-de-la-Camp-Platz 1, 44789 Bochum, Germany; 4 Seattle Science Foundation, 550 17th Avenue, Suite 600, Seattle, WA 98122, USA; 5 Department of Orthopedics and Trauma Surgery, St. Josef Hospital Essen-Werden, Propsteistrasse 2, 45239 Essen, Germany

**Keywords:** spondylodiscitis, readmission rate, spine location, spine infection, risk factor

## Abstract

**Background/Objectives**: Primary spondylodiscitis (SD) cases surging in incidence globally remain a diagnostic and therapeutic challenge for physicians. The effect of lesion location on outcomes remains unclear. This study aims to assess the 90-day all-cause readmission rate in patients suffering from spondylodiscitis in different regions of the spine, with a secondary objective of comparing in-hospital mortality rates. **Methods**: Utilizing the 2020 Nationwide Readmissions Database (NRD), USA, adult patients (>18 years) were selected by diagnosis with ICD-10 codes for primary spondylodiscitis. Patients were categorized by localization into eight groups, excluding multifocal patients. Comparative analysis and logistic regressions were performed. **Results**: Among 5547 patients, lumbar SD was most prevalent, followed by thoracic and lumbo-sacral regions. Cervical SD had the lowest readmission rate (31.3%) and lower odds versus lumbar SD (adjusted OR = 0.73; *p* = 0.007). Other regions showed no significant differences. In-hospital mortality varied by location. The sacral region, renal failure, and advanced age were the strongest mortality predictors. **Conclusions**: While the incidence of spondylodiscitis varies by location on the spine, we found no significant differences in readmission rates across regions. However, there were substantial differences in in-hospital mortality rates. Comorbidities, particularly renal failure and advanced age, appear to outweigh spinal localization as risk factors for mortality and readmission.

## 1. Introduction

De novo spondylodiscitis has been found to be a rapidly increasing disease entity globally. Factors such as an aging population, rising prevalence of comorbidities such as immunosuppression and intravenous drug use, and improvements in diagnostic imaging resources all play a likely role in this development [1,2,3,4]. A large epidemiological study in England reported an increase of up to 44% in less than 10 years [5]. In addition to its rising incidence, spondylodiscitis continues to present a diagnostic and therapeutic challenge due to its relatively non-specific presenting symptoms, such as fever, back pain, and, occasionally, neurological manifestations. Despite considerable advancements in imaging, ongoing challenges in SD care revolve around timely diagnosis and the burden of morbidity and mortality. Established risk factors for increased early mortality include diabetes, a history of steroid use, and advanced age [6,7]. Although recent studies have sought to formally standardize the diagnosis and treatment of spinal infections, such as through the development of the SITE score, management of spinal column infections remains a challenge [3,8,9]. Further complicating management of spondylodiscitis is the variability in occurrence and morbidity associated with different anatomic regions of the spine [10,11,12]. To our knowledge, readmission and mortality rates for spondylodiscitis relative to different regions of the spinal column have not yet been investigated. The primary objective of this study was to assess the 90-day all-cause readmission rate in patients suffering from spondylodiscitis across different areas of the spine, with the secondary objective of comparing in-hospital mortality rates in these patients.

## 2. Materials and Methods

### 2.1. Study Design and Data Source

A retrospective cohort study was performed using the 2020 Nationwide Readmissions Database (NRD), part of the Healthcare Cost and Utilization Project (HCUP), Agency for Healthcare Research, USA. This database comprises datasets of hospital discharge records from participating states in the United States and provides a nationally weighted and stratified sample.

We extracted clinical and admission data, including demographic information, length of stay (LOS), in-hospital death, as well as comorbidities.

Comorbidities present at discharge were identified using ICD-10 codes and the primary Clinical Classifications Software Refined (CCSR) for International Classification of Diseases (ICD)-10 category codes, Version 2023.1. As a measure of severity, the Elixhauser Comorbidity Index for in-hospital mortality and all-cause 90-day readmission were calculated for each patient [13]. In accordance with the HCUP data use agreement, any variables were excluded from tabulated comparison, if numbers were 10 or fewer. No institutional review board approval was needed for this project, as the data are publicly available.

### 2.2. Population

Adult patients (>18 years) coded as spondylitis diseased (M46.2x, M46.3x, and M46.4x) using International Classification of Diseases (ICD)-10 codes, Version 2023.1 were included. Exclusion criteria included the presence of malignancy, traumatic injuries, secondary spinal column infections, and incomplete datasets. Patients with infections involving multiple regions of the spine were also excluded from this analysis. The 90-day all-cause readmission rate was calculated by only including patients treated within the first 9 months of 2020. The regions of the spine were divided into 8 different groups by their anatomical location.

### 2.3. Outcomes

The cohorts were categorized into 2 groups based on their readmission status, as identified by NRD VisitLinks. Time to readmission was defined as the number of days between a patient’s discharge and their subsequent admission to the hospital. In-hospital mortality was defined as the death of a patient during their hospital stay, thus capturing only early mortality rates.

### 2.4. Statistical Analysis

Frequency counts were computed and presented along with their percentages for descriptive statistics. For continuous variables, means were computed and presented along with their standard deviations. Bivariable analysis was performed to compare readmission rates between treatment groups, using Chi-square tests for categorical variables and logistic regression for continuous variables. To evaluate the effect of spondylodiscitis location on readmission and in-hospital mortality rates, we performed a multivariable logistic regression analysis, with the lumbar region as the reference group. Potential confounding factors were assessed for collinearity. Factors included in the multivariable analysis were based on clinical expert opinion and the relevant literature. Analyses were performed using Stata software, version 15.0 (College Station, TX, USA).

## 3. Results

### 3.1. Demographics

A total of 5547 patients met our inclusion criteria. The majority of spinal infections were localized in the lumbar region (n = 2414), followed by the thoracic (n = 1120) and sacral (n = 884) regions (Table 1).

The average age varied across groups, ranging from 55.90 ± 15.29 years in the lumbo-sacral group to 65.16 ± 15.17 years in the thoraco-lumbar group. These differences were statistically significant (*p* ≤ 0.001). Across all groups, male patients were in the majority, with the cervico-thoracal region showing the highest percentage (75.00%) and the sacral region the lowest (49.21%; *p* < 0.001). The Elixhauser indices revealed notable significant differences in comorbidity burdens. The in-hospital mortality index was highest for cervico-thoracal infections (1.78 ± 9.52) and lowest for lumbo-sacral infections −1.54 ± 9.07; *p* < 0.001). Similarly, the 30-day readmission index was highest for cervico-thoracal infections (6.72 ± 5.93) and lowest for locations “not specified” (4.12 ± 5.68; *p* = 0.293). Most admissions were non-elective, with rates above 93% in every group except for “other” (87.88%; *p* = 0.699). The LOS was significantly longer for the cervico-thoracal group (22.44 ± 42.47 days) compared to other regions, such as lumbar (9.89 ± 9.89 days) or “not specified” (8.44 ± 10.04 days; *p* = 0.019).

The prevalence of comorbidities varied significantly among groups. Hypertension was the most common comorbidity, with 69.70% affected in the “not specified” group (*p* < 0.001). Diabetes mellitus was also prevalent, ranging from 19.7% in “not specified” to 34.38% in cervico-thoracal infections (*p* < 0.001). We also observed significant differences between the groups for obesity, depression, autoimmune diseases, renal failure and chronic lung diseases.

### 3.2. Outcomes

Readmission rates were similar and did not show significant differences across all groups (Table 2). Rates ranged from 31.3% in cervical infections to 46.88% in the cervico-thoracal region (*p* = 0.387). However, the time to readmission varied significantly between groups (*p* = 0.0026). Cervico-thoracal infections had the longest average time to readmission at 42.47 ± 27.60 days, followed by sacral infections at 41.07 ± 22.73 days. In contrast, “not specified” regions demonstrated the shortest time to readmission at 29.89 ± 23.47 days.

In-hospital mortality rates were low across all regions and varied significantly (*p* < 0.001). Due to the relatively low in-hospital mortality rate, reflecting only early events, most values fell below the reporting minimum of 11 cases and could not be included in the tabulation. The lumbar group had a mortality rate of 1.04%, while the sacral group had the highest mortality rate at 2.83%.

### 3.3. Multivariable Analysis Evaluating the Effect of Location on Readmission Rates

The multivariable analysis showed that spinal infection location had a variable impact on readmission rates (Table 3). Cervical infections were associated with a significantly reduced risk of readmission compared to the reference group (aOR 0.73, 95% CI: 0.58–0.93, *p* = 0.007). No other spinal regions included in the analysis showed statistically significant differences in readmission risk.

We found diverse effects of patient factors on readmission rates. Age was inversely associated with readmission, albeit with a small effect size (aOR 0.99, 95% CI: 0.99–0.99, *p* < 0.001), while male gender was associated with a slight increase in readmission risk (aOR 1.14, 95% CI: 1.01–1.29, *p* = 0.028).

Comorbidities had mixed effects. Thyroid disease was the strongest predictor of readmission (aOR 3.62, 95% CI: 3.11–4.21, *p* < 0.001). Heart failure (aOR 1.88, 95% CI: 1.57–2.24, *p* < 0.001), hypertension (aOR 1.22, 95% 1.07–1.40, *p* < 0.001), and renal failure (aOR 1.85, 95% CI: 1.51–2.35, *p* < 0.001) were also associated with an increased risk of readmission. Conversely, obesity showed a reduced risk of readmission (aOR 0.85, 95% CI: 0.72–1.00, *p* = 0.047). Other factors, such as diabetes mellitus and autoimmune diseases, did not show statistically significant associations with readmission risk in the adjusted model.

### 3.4. Multivariable Analysis Evaluating the Effect of Location on In-Hospital Mortality

The multivariable analysis identified significant predictors of in-hospital mortality (Table 4). The sacral group were strongly associated with an increased risk of in-hospital mortality (aOR 3.15, 95% CI: 1.75–5.69, *p* < 0.001), while the thoraco-lumbar region showed a trend toward higher mortality risk, but this did not reach statistical significance (aOR 2.62, 95% CI: 0.97–7.11, *p* = 0.059). Age (aOR 1.07, 95% CI: 1.05–1.09, *p* < 0.001) and renal failure (aOR 3.40, 95% CI: 1.77–6.53, *p* < 0.001) were also identified as individual risk factors. Interestingly, thyroid disease was inversely associated with in-hospital mortality (aOR 0.47, 95% CI: 0.22–0.99, *p* = 0.048). Other comorbidities, such as hypertension, diabetes, and chronic lung disease, did not show statistically significant associations with mortality in the adjusted model. However, the “not specified” region and liver failure were excluded from the analysis due to perfect prediction of mortality, resulting either from the absence of events or from the occurrence of death in all affected patients.

## 4. Discussion

The global incidence of spondylodiscitis continues to rise, driven primarily by an aging population, increased intravenous drug use, and a rise in comorbidity burden [1,2,3]. Given the global increase in incidence of spondylodiscitis and ongoing challenges of resource utilization in socioeconomically distressed healthcare systems, an improved understanding of variables that affect spondylodiscitis outcomes in terms of morbidity and mortality rates would be helpful to guide management and risk assessment strategies. The impact of anatomic regions on patient outcomes has not been scientifically explored in this context, and their influence on readmission rates and mortality remains unclear.

In our analysis, we found that the lumbar spine was the most commonly affected area of all the anatomic regions of the spine, with a total of 2414 patients, a finding largely consistent with prior studies [1,14,15]. While there is not a definitive explanation for this dominance, greater vascularity of the lumbar spinal segments and higher mechanical stress load in comparison to other regions of the spine may account for this finding [10,16].

Our readmission rate showed no statistically significant variation between the different regions, with the cervical spine region having the lowest rate and the cervico-thoracal group the highest (31.3% vs. 46.88%). In contrast to this, the “not specified” region experienced the shortest time to readmission and the cervico-thoracal the longest (29.89 ± 23.47) vs. (42.47 ± 27.60). Compared to other spinal pathologies, these readmission rates remain notably higher. For spine surgery and spinal diseases, reported 90-day all-cause readmission rates vary widely, reflecting differences in morbidity, severity, and the complexity of disease management. For example, patients treated for elective, degenerative lumbar spine surgery have an unplanned readmission risk of 7.71%, as reported in a recent meta-analysis. Interestingly, the timeframes until readmission are comparable across these groups [17,18,19]. The discrepancies between our 90-day all-cause readmission rates and those reported in other spinal conditions may be explained by several factors. First, spondylodiscitis may be more aggressive and difficult to manage than degenerative spinal conditions. SD patients require prolonged intravenous antibiotics, carry higher risks of systemic complications such as sepsis or endocarditis, and often warrant multiple interventions. Furthermore, patients hospitalized and treated for metastatic spine diseases experience rates of up to 35.7% [20]. Although this rate is within range of our results, SD appears to impose a greater burden on patients and necessitates a greater use of healthcare resources. Overall, the much higher readmission rate associated with SD underscores the severity of the disease and the complexity of its management in these patients.

Although we found no significant difference in overall readmission rates across spinal regions, the cervical region was associated with decreased odds of readmission compared to the lumbar spine (aOR = 0.73, *p*-value = 0.007), while the cervico-thoracal region had the highest rate of readmission. This is a paradoxical finding, given the increased risk in the cervical spine of post-infectious deformity, neurological deficits, and other morbidity and mortality measures compared to other regions [21,22]. This finding may be explained by the tendency toward more aggressive and definitive surgical intervention in cervical spine infections, with more surgical interventions for spondylodiscitis performed in the cervical spine compared to other regions [12,21]. In addition to this, surgical treatment in patients suffering from spondylodiscitis have been shown to significantly reduce the risk of readmission. In contract to this, patients treated non-surgically had a significantly higher readmission risk with a shorter time until readmission [23]. Additionally, the cervical region did not exhibit increased odds for in-hospital mortality or LOS (11.15 days ± 11.23), further suggesting potential benefits from early and decisive surgical management. This could be confounded by a relatively low comorbidity burden in the cervical spine group, as reflected by the Elixhauser indices, compared to the other groups.

The highest in-hospital mortality rates among spinal regions, based on multivariable analysis, were the lumbar and sacral regions (1.04% and 2.83%, respectively). Sacral infections, in particular, present unique clinical challenges due to limited surgical accessibility, complex pelvic anatomy, delayed recognition and diagnosis, and their association with pressure ulcers and sacral osteomyelitis [24]. Multivariable logistic regression analysis identified the sacral region (aOR = 3.15, *p* < 0.001), age (aOR = 1.07, *p* ≤ 0.001), and renal failure (aOR = 3.40, *p* < 0.001) as among the strongest predictors of in-hospital mortality.

The effect of the anatomic region of spondylodiscitis on mortality has not received enough attention; the only previously published study exploring mortality differences across spinal regions was a single-center study with a cohort of 211 patients. This study did not find statistically significant differences in mortality among spine regions [10]. The discrepancy between our study results and the previous study may be reflected in differences in sample sizes, patient demographics, healthcare settings, and methodological approaches. Interestingly, in our study, thyroid disease was associated with a reduced risk of mortality (aOR = 0.47, *p* = 0.048), though the underlying mechanisms for this finding remain unclear and warrant further investigation. Overall, our results suggest that certain comorbidities and advanced age play a more significant role than spinal localizations regarding in-hospital mortality in patients with spondylodiscitis.

It is well established that an increasing number and variety of comorbidities affect the development of spondylodiscitis, as well as its readmission and mortality rates [1,2,5,7,25]. As suggested by Gerstmeyer et al., the risk factors for the development of spondylodiscitis differ; our analysis highlights notable variations in comorbidity burdens among the groups, as reflected by the Elixhauser indices. Specifically, the cervico-thoracic region emerged with the highest comorbidity burden and longest LOS, potentially due to the junctional location, which introduces complexity in management.

Hypertension was the most common comorbidity, consistent with prior studies [26,27]. Diabetes mellitus was also common and most frequent among the cervico-thoracic group (34.38%). This is consistent with previous findings reporting higher rates of spondylitis in patients with obesity and diabetes mellitus [28]. These results confirm the overall importance of risk factor management in the development of spondylodiscitis. Unsurprisingly, severe conditions like heart failure, renal failure, hypertension, and thyroid disease were associated with increased risks of readmission (aOR of 1.88, 1.85, 1.22, and 3.62, respectively; *p* < 0.001), given the chronicity of these comorbidities. Interestingly, there was a significantly decreased risk of readmission with obesity (aOR = 0.85, *p* = 0.047). This finding seems counterintuitive, as obesity is a well-established risk factor for severe disease causes [28].

The only strong predictor of in-hospital mortality in our study was renal failure. Despite the known infection-related complications of diabetes mellitus, we found no significantly increased odds for either readmission or mortality. These findings confirm that disease-related risk factors differ from those related to readmissions and mortality, especially early in-hospital mortality [7,29]. The severity of SD and its causative pathogen have also been shown to influence mortality rates [7,30]. However, the effect of pathogens on readmission rates remains to be determined.

While we provide valuable information regarding risk factors for morbidity and mortality for patients suffering from primary spondylodiscitis across various regions of the spinal column, there are unmodifiable limitations inherent to the use of databases as tools for clinical research. The multidimensional complexity of the disease—particularly its actual extent and severity, as reflected in the severity of bony destruction, the specifics and duration of antibiotic treatment, and the identity of the causative pathogen—is unlikely to be fully captured in a generic database. A more comprehensive analysis is constrained by the absence of detailed information and the reliance on data available at discharge.

Furthermore, the data were extracted in 2020 during the COVID-19 pandemic, a period in which hospital admission and treatment protocols may have deviated from non-pandemic times. We did not include the modality of treatment (surgical vs. non-surgical), as this was analyzed in an independent study. Treatment and outcomes in patients suffering from primary spondylodiscitis may be influenced by a multitude of variables, not all of which are captured in this analysis. Lastly, large database analyses are susceptible to Type 1 errors, which may detect modest, non-clinically meaningful associations.

Future studies may further explore the relationships identified in our analysis by including more detailed clinical data.

## 5. Conclusions

Despite overall high readmission rates in cases of primary spondylodiscitis, we found no significant differences when comparing anatomic spinal regions. However, in-hospital mortality rates differed significantly, with the highest in-hospital mortality occurring with infections in the sacral region. Overall, our findings indicate that comorbidities, specifically renal failure, heart failure, and advanced age, may have a greater impact on mortality and readmission outcomes than specific spinal localization. These results underline the importance of comprehensive patient management strategies that address both the infectious source and associated comorbidities. Future research aimed at clarifying these associations would be beneficial to enhancing treatment for patients with primary spondylodiscitis.

## Figures and Tables

**Table 1:** Demographics and comorbidities.

	Cervical N = 467	Cervico-Thoracal N = 32	Thoracal N = 1120	Thoraco-Lumbar N = 157	Lumbar N = 2414	Lumbo-Sacral N = 407	Sacral N = 884	Not Specified N = 66	*p*-Value
				**N (%) or**	**Mean (± SD)**				
**Patient demographics**									
Age (years)	57.36 ± 13.83	61.06 ± 11.42	58.96 ± 14.84	65.71 ± 15.44	61.51 ± 15.53	55.90 ± 15.29	61.28 ± 18.72	59.56 ± 16.03	**<0.001**
Male	302 (64.67)	24 (75.00)	684 (61.07)	89 (56.69)	1502 (62.22)	247 (60.69)	435 (49.21)	41 (62.12)	**<0.001**
Elixhauser in-hospital mortality index	–0.34 ± 9.63	1.78 ± 9.52	–0.34 ± 9.03	0.24 ± 10.41	–0.72 ± 9.27	–1.54 ± 9.07	0.39 ± 9.24	0.59 ± 8.28	**<0.001**
Elixhauser 30-day readmission index	5.94 ± 6.12	6.72 ± 5.93	5.74 ± 5.64	6.05 ± 5.78	5.50 ± 5.61	5.09 ± 5.26	4.78 ± 5.30	4.12 ± 5.68	0.293
**Admission details**									
Non-elective admission	438 (93.79)	30 (93.75)	1050 (93.75)	147 (93.63)	2266 (93.87)	382 (93.86)	827 (93.55)	58 (87.88)	0.699
Length of stay (days)	11.15 ± 11.23	22.44 ± 42.47	11.53 ± 12.47	11.59 ± 15.01	11.20 ± 13.58	9.89 ± 9.89	12.39 ± 18.35	8.44 ± 10.04	**0.019**
**Comorbidities**									
Obesity	55 (11.78)	*	182 (16.25)	24 (15.29)	412 (17.07)	56 (13.76)	125 (14.14)	11 (16.67)	0.149
Diabetes mellitus type 2	124 (26.55)	11 (34.38)	359 (32.05)	53 (33.76)	732 (30.32)	86 (21.13)	197 (22.29)	13 (19.70)	**<0.001**
Hypertension	276 (59.10)	21 (65.63)	727 (64.91)	109 (69.43)	1575 (65.24)	221 (54.30)	521 (58.94)	46 (69.70)	**<0.001**
Depression	56 (11.99)	*	152 (13.57)	22 (14.01)	358 (14.83)	66 (16.22)	120 (13.57)	10 (15.15)	0.496
Autoimmune disease	24 (5.14)	*	40 (3.57)	10 (6.37)	108 (4.47)	21 (5.16)	23 (2.60)	*	**0.047**
Chronic lung disease	87 (18.63)	*	240 (21.43)	25 (15.92)	443 (18.35)	63 (15.48)	133 (15.05)	*	**0.015**
Thyroid disease	98 (20.99)	*	174 (15.54)	22 (14.01)	363 (15.04)	61 (15.00)	178 (20.14)	*	**<0.001**
Heart failure	60 (12.85)	*	165 (14.73)	29 (18.47)	349 (14.46)	42 (10.32)	109 (12.33)	*	**0.040**
Renal failure	48 (10.28)	*	95 (8.48)	12 (7.64)	170 (7.04)	21 (5.16)	29 (3.28)	*	**<0.001**
Liver failure	*	*	*	*	*	*	*	*	0.945

Bold variables indicate statistical significance. * Indicates that value was below Healthcare Utilization Project reporting minimum of 11, due to privacy protection guidelines.

**Table 2:** Readmission and in-hospital mortality rates.

	Cervical N = 467	Cervico-Thoracal N = 32	Thoracal N = 1120	Thoraco-Lumbar N = 157	Lumbar N = 2414	Lumbo-Sacral N = 407	Sacral N = 884	Not Specified N = 66	*p*-Value
				N (%) or	Mean (± SD)				
Readmission	146 (31.3)	15 (46.88)	392 (35.0)	55 (35.03)	839 (34.8)	139 (34.14)	324 (36.65)	28 (42.42)	0.387
Time to readmission (days)	35.33 ± 23.85	42.47 ± 27.60	34.68 ± 23.14	34.87 ± 24.68	34.50 ± 22.05	33.02 ± 22.94	41.07 ± 22.73	29.89 ± 23.47	**0.002**
In-hospital mortality	*	*	*	*	25 (1.04)	*	25 (2.83)	*	**<0.001**

Bold variables indicate statistical significance. * Indicates that value was below Healthcare Utilization Project reporting minimum of 11, due to privacy protection guidelines.

**Table 3:** Multivariable logistic regression analysis estimating the effects on readmission.

Factor	aOR	95% CI	*p*-Value
Cervical	0.73	0.58–0.92	**0.007**
Cervico-thoracal	1.53	0.74–3.18	0.256
Thoracal	0.96	0.83–1.13	0.646
Thoraco-lumbar	1.05	0.74–1.49	0.794
Lumbo-sacral	0.97	0.77–1.23	0.824
Sacral	1.08	0.91–1.28	0.371
Not specified	1.48	0.88–2.47	0.136
**Covariates**			
Age (years)	0.99	0.99–0.99	**<0.001**
Male	1.14	1.01–1.29	**0.028**
Autoimmune disease	0.80	0.59–1.08	0.139
Depression	1.14	0.97–1.34	0.115
Chronic lung disease	1.11	0.96–1.29	0.153
Obesity	0.85	0.72–1.00	**0.047**
Heart failure	1.88	1.57–2.24	**<0.001**
Thyroid disease	3.62	3.11–4.21	**<0.001**
Hypertension	1.22	1.07–1.40	**0.004**
Diabetes mellitus type 2	1.00	0.87–1.14	0.958
Liver failure	2.25	0.47–10.81	0.312
Renal failure	1.85	1.47–2.33	**<0.001**

Bold variables indicate statistical significance.

**Table 4:** Multivariable logistic regression analysis estimating the effects on in-hospital mortality.

Factor	aOR	95% CI	*p*-Value
Cervical	1.42	0.53–3.81	0.484
Cervico-thoracal	3.83	0.48–30.76	0.206
Thoracal	0.78	0.35–1.75	0.543
Thoraco-lumbar	2.62	0.97–7.11	0.059
Lumbo-sacral	1.06	0.31–3.57	0.927
Sacral	3.15	1.75–5.69	**<0.001**
Age (years)	1.07	1.05–1.09	**<0.001**
Male	1.32	0.80–2.19	0.277
Autoimmune disease	0.32	0.04–2.38	0.268
Depression	1.14	0.59–2.22	0.694
Chronic lung disease	0.99	0.53–1.84	0.975
Obesity	0.65	0.31–1.35	0.247
Heart failure	1.49	0.83–2.66	0.181
Thyroid disease	0.47	0.22–0.99	**0.048**
Hypertension	0.78	0.42–1.45	0.430
Diabetes mellitus type 2	1.60	0.96–2.68	0.071
Renal failure	3.40	1.77–6.53	**<0.001**

The reference category for the analysis was the lumbar region. Other spinal regions and liver failure were excluded from the analysis because they perfectly predicted mortality. Bold variables indicate statistical significance.

## Data Availability

The dataset is publicly available from the Healthcare Cost and Utilization Project (HCUP), Agency for Healthcare Research and Quality.

## Abbreviations

The following abbreviations are used in this manuscript:

SD	Spondylodiscitis
ICD-10	International Classification of Diseases, 10th Revision
NRD	Nationwide Readmissions Database
HCUP	Healthcare Cost and Utilization Project
CCSR	Clinical Classifications Software Refined
LOS	Length of Stay
aOR	Adjusted Odds Ratio
CI	Confidence Interval
SITE	Spinal Infection Treatment Evaluation Score
COVID-19	Coronavirus Disease 2019
